# SARS-CoV-2 Infection and Outcomes in Children with Inflammatory Bowel Diseases: A Systematic Review

**DOI:** 10.3390/jcm11237238

**Published:** 2022-12-06

**Authors:** Anastasia Batsiou, Petros Mantzios, Daniele Piovani, Andreas G. Tsantes, Paschalia Kopanou Taliaka, Paraskevi Liakou, Nicoletta Iacovidou, Argirios E. Tsantes, Stefanos Bonovas, Rozeta Sokou

**Affiliations:** 1 Neonatal Intensive Care Unit, “Agios Panteleimon” General Hospital of Nikea, 18454 Piraeus, Greece; 2 Department of Internal Medicine, General Hospital of Eastern Achaia, 25100 Aigio, Greece; 3 IRCCS Humanitas Research Hospital, 20089 Milan, Italy; 4 Department of Biomedical Sciences, Humanitas University, 20090 Milan, Italy; 5 Microbiology Department, “Saint Savvas” Oncology Hospital, 11522 Athens, Greece; 6 Neonatal Department, Aretaieio Hospital, National and Kapodistrian University of Athens, 11528 Athens, Greece; 7 Laboratory of Haematology and Blood Bank Unit, “Attiko” Hospital, School of Medicine, National and Kapodistrian University of Athens, 12462 Athens, Greece

**Keywords:** inflammatory bowel diseases, COVID-19, SARS-CoV-2, child, pediatrics

## Abstract

The impact of COVID-19 on pediatric patients with inflammatory bowel disease (PIBD) is still not clear and the knowledge acquired over the last 2 years is still evolving. This study aims to investigate the risk and clinical outcomes of COVID-19 in patients with PIBD. A systematic search of PubMed and Scopus databases was conducted to identify studies published up until September 2022. Out of the 475 articles screened, 14 studies were included in the review. Of the 4006 children with PIBD included, 390 (9.7%) tested positive for COVID-19. Among those with COVID-19, 5.9% (0–16.7%) needed hospitalization, 0.6% (0–1%) were admitted to the pediatric intensive care unit (PICU), and no deaths were reported. Among the included studies, only four presented details regarding patients’ symptoms, with 21% (0–25%) presenting gastrointestinal (GI) symptoms. An association between PIBD activity or specific treatment and COVID-19 outcome could not be established. The prevalence of COVID-19 in patients with PIBD was low; therefore, the initial concerns regarding higher infection risk and worse prognosis in this population are not supported by the currently available data. Further research is needed to determine the natural history of the infection and the optimal treatment for these patients. Much is still unclear and additional studies should be performed in order to optimize prevention and care for this special group of patients.

## 1. Introduction

COVID-19 has emerged as one of the most devastating infectious diseases of the 21st century. It is caused by the SARS-CoV-2 virus and has affected more than 623 million individuals, with more than 6.5 million reported deaths until November 2022 [1]. However, most infected patients experience only mild to moderate symptoms and recover without any specific treatment [2].

Pediatric patients with COVID-19 experience milder disease of shorter duration with fewer complications compared to adults. According to a meta-analysis conducted by Mantovani et al. [3], the most common presentation in this age group includes fever, cough, and rarely diarrhea. Although the prognosis is generally good, a new hyperinflammatory syndrome affecting children with recent SARS-CoV2 infection, termed multisystem inflammatory syndrome in children (MIS-C), has raised serious concerns [4].

Inflammatory bowel diseases (IBD) are chronic relapsing autoimmune disorders that affect the gastrointestinal tract, including Crohn’s disease (CD), ulcerative colitis (UC), and IBD-unspecified (IBD-U) [5]. Over the last 25 years, a steadily increasing incidence of pediatric-onset IBD (PIBD) has been reported in various studies, with children and adolescents accounting for 25% of the total number of patients [6,7]. Multifactorial dysregulation of the immune system targeting the gut mucosa and intestinal microbiota in genetically predisposed patients is the underlying mechanism involved in the pathogenesis of IBD. Treatment of IBD is based on the regulation of the over-reacting immune system which is usually achieved through immunomodulators such as monoclonal therapy, biologic agents, and steroids [8]. Anti-tumor necrosis factor (anti-TNF) is one of the most common agents administered in young patients, with its use rising from 27.1% in 2017 to 44% in 2021 [7]. Given a more severe disease phenotype, patients with PIBD are more likely to receive thiopurines as compared to adults. Hence, these are usually immunosuppressed patients with a potential higher risk for COVID-19 and severe disease. However, the actual risk of patients with IBD receiving immunomodulators and immunosuppressant therapy remains unclear.

The Surveillance Epidemiology of Coronavirus Under Research Exclusion for Inflammatory Bowel Disease (SECURE-IBD) was established to monitor patients with IBD and COVID-19 in order to gather more information regarding the risk and the course of infection in these patients [7]. There is a growing body of evidence regarding COVID-19 in adults with IBD, indicating that IBD is not associated with a higher infection risk or a worse prognosis [9]. Moreover, there is no data supporting that COVID-19 is associated with IBD flares in adult patients. There is only sparse information regarding the clinical course and outcome of COVID-19 in PIBD patients.

The purpose of this study was to review and analyze the available epidemiologic data regarding COVID-19 in pediatric IBD patients, in order to evaluate the risk of COVID-19 and its clinical outcome in children with IBD.

## 2. Materials and Methods

### 2.1. Search Strategy—Databases

A systematic review methodology following the guidelines of Preferred Reporting Items for Systematic Reviews and Meta-Analyses (PRISMA) [10,11] was chosen in order to excerpt, evaluate, and interpret the published studies related to our research objective.

We searched PubMed and Scopus databases from inception to 30 September 2022. The following keywords were used: “coronavirus disease 19 “, “COVID-19”, “SARS-CoV-2”, “inflammatory bowel disease”, “ulcerative colitis”, “Crohn’s disease”, “IBD”, “UC”, “CD”, “Child *”, “children *”, “pediatric patient *”, “infant *”, “adolescent *”, with Boolean logical operators.

Furthermore, in order to decrease the risk of missing relative studies, a manual electronic search and review of the references of each retrieved study was performed. References of already published reviews on the same research subject were also scanned. Studies published in languages other than English, as well as editorials, letters to editor, and conference papers, were excluded. No geographic restrictions were imposed.

### 2.2. Selection Criteria

Any study meeting the following criteria was included: (1) any pediatric patient with confirmed diagnosis of IBD and (2) any patient in the study population with confirmed COVID-19 diagnosis with a positive diagnostic test. We intended to screen all articles reporting COVID-19 cases in children and adolescents with PIBD. We specifically focused on each article describing the proportion of patients with PIBD developing COVID-19 (i.e., positive throat swab for SARS-CoV-2) and/or the management of pediatric participants and morbidity/recovery/mortality rate, as well as complications attributed to the administered medications. Studies enrolling both adults and children were included, if adequate information was available regarding pediatric participants separately.

Narrative reviews, systematic reviews, meta-analyses and studies that reported data based solely on adults (older than 18 years old) were excluded.

### 2.3. Study Selection and Data Extraction

Four authors (A.B., N.I., A.G.T., and P.M.) individually screened relevant titles and abstracts according to the inclusion and exclusion criteria, and a thorough full text evaluation was followed (A.B., P.M., A.E.T., P.K.T., P.L., D.B., and S.B.) after exclusion of the clearly irrelevant articles. We extracted from each study the proportion of patients with PIBD who tested positive for COVID-19 and required hospitalization or pediatric intensive care unit (PICU) admission; demographic data, clinical parameters, and information on treatment and complications were recorded. Any disputes between the researchers regarding selection of the studies were resolved through discussion or with the contribution of another author (R.S.).

## 3. Results

### 3.1. Study Characteristics

**Figure 1:**
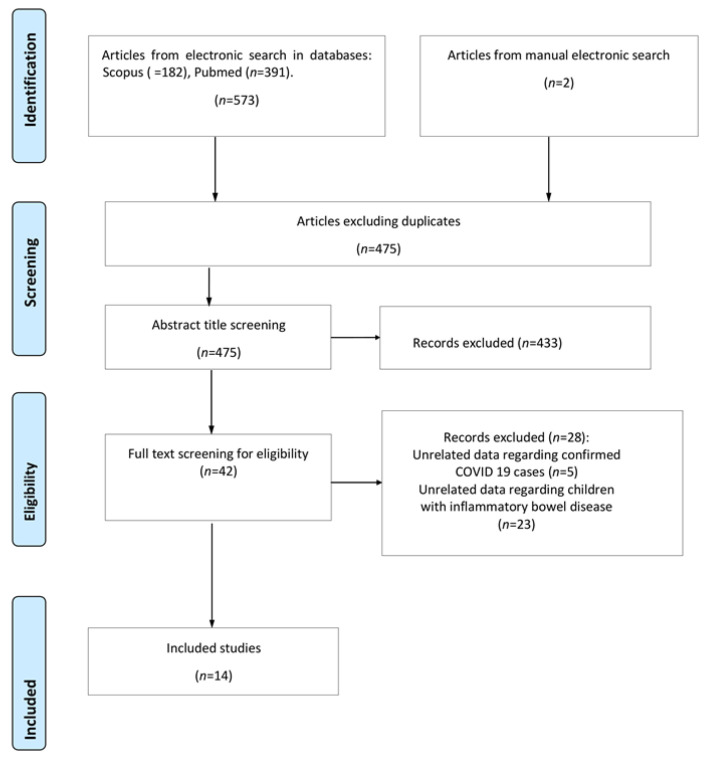
Flow chart of the selection process.

The initial electronic database search retrieved 575 studies, 100 of which were duplicates. Duplicate records were removed by a single researcher (R.S.) using the default settings in EndNote X8 (Clarivate, Philadelphia, PA, USA). Following screening of the titles and abstracts of the remaining studies, 433 were excluded either because they were irrelevant to the posed research question or because they met some of the exclusion criteria. Only 14 of the 42 remaining studies were eligible for inclusion in this review, after meticulous full text evaluation. The flow diagram of the study selection is depicted in Figure 1.

Of the 14 studies included in our systematic review, 12 were observational studies [8,12,13,14,15,16,17,18,19,20,21,22] (9 cohort studies, 2 cross-sectional studies, and 1 case-control study), while the other 2 were case report/series [23,24]. All studies included patients with PIBD with adequate data about SARS-CoV-2 infection. In certain studies, the population included adults, children, and adolescents; however, specific and well-defined data regarding pediatric patients were provided [14,15,17,18,20]. Unfortunately, due to many plausible reasons for extreme heterogeneity such as different aims of the included studies (and consequently inclusion and exclusion criteria), different populations, and different medical treatment used for IBD across studies (but absence of individual patient data to take this important information into account), a meta-analysis was not deemed appropriate.

### 3.2. IBD Disease and COVID-19

Our research yielded 14 studies with data on the incidence of COVID-19 in patients with PIBD (Table 1).

From a total of 4006 included children with PIBD, 390 (9.7%) tested positive for COVID-19. The data regarding the clinical course of COVID-19 were reported for 339 patients, out of whom 296 (87.3%) did not need hospitalization, 20 (5.9%) were admitted to the hospital, and 2 (0.6%) to the pediatric intensive care unit (PICU), with a reported range across studies of 83–100%, 0–16.7%, and 0–1%, respectively (Table 2). No deaths were recorded.

Among the studies, only four presented details on the exact symptoms experienced by patients with PIBD during COVID-19 infection [12,13,15,24]. From a pool of 235 patients with PIBD who tested positive for SARS-CoV-2, 21% presented gastrointestinal symptoms, with a large variability across the studies (0–25%). Specifically, 3.8% (0–4%) exhibited vomiting, 6% (0–8%) nausea, 12.2% (0–17%) diarrhea, and 11.8% (0–13%) abdominal pain. Arrigo et al. [12], in a multicenter, retrospective study, reported data on 2291 children with PIBD in 21 IBD referral centers. Surprisingly, only six cases tested positive for SARS-CoV-2, out of whom two suffered from CD and four from UC. Only one patient needed hospitalization but eventually recovered.

Brenner et al. [14] investigated the impact of COVID-19 on patients with PIBD, based on the SECURE-IBD. They reported 29 PIBD cases, all exhibiting mild symptoms, out of which only 3 needing hospitalization and none required admission to PICU.

As presented by Martinelli et al. [21], in a cohort of 180 children with IBD (UC = 72, CD = 108) followed-up during the lockdown, a significant reduction (up to 64.2%) in IBD flare-related hospital admissions was reported, compared to the 2-month period prior to the pandemic, possibly indicating prioritization in reducing social contact over seeking timely medical treatment. No confirmed COVID-19 cases were registered [21].

A global study conducted by the Paediatric IBD Porto group of European Society for Paediatric Gastroenterology Hepatology and Nutrition (ESPGHAN), that was published in June 2020, provided a significant literature update on the overall effect of the novel COVID-19 pandemic on the treatment of patients with PIBD. In this study, Turner et al. [24] reported the first eight confirmed COVID-19 cases in patients with PIBD, with five, two, and one having CD, UC, and IBD-U, respectively. In this group of patients, the viral disease presented with mild symptoms, mainly cough (*n* = 4), low grade fever (*n* = 3), and fatigue (*n* = 3). No hospitalization was required, and no death was reported, despite the fact that these patients were receiving immunomodulatory treatments.

Brenner et al. [13] included 209 patients with PIBD and COVID-19 and noted that only 7% needed admission to the hospital and only 2 of them presented with severe symptoms which led to their admission in the PICU. A total of 50% of the hospitalized cases had comorbidities. A total of 66% (*n* = 138) of patients had CD, 29% (*n* = 61) had UC, and the rest had IBD-U. In 23% (*n* = 47) of the cases, the gastrointestinal system was affected with various symptoms such as abdominal pain and diarrhea. D’Arcangelo et al. [16], in a retrospective cohort study, included 185 patients with PIBD out of whom 4 tested positive for SARS-CoV-2. Two (2) of them were asymptomatic and the remaining exhibited only mild symptoms.

Koletzko et al. [17] investigated the impact of COVID-19 on patients with IBD and identified a total of 25 cases of confirmed infection in a total of 89 patients with PIBD, none of which required hospitalization. Queiroz et al. [18] reported outcomes of patients from Latin America enrolled in the SECURE-IBD registry, including 18 children infected with the virus, with only 1 of them requiring hospitalization but without admission to the PICU. Ruan et al. [8], in a single-center cohort study, reported 14 patients with PIBD and COVID-19 (10 diagnosed with CD and 4 with UC). Seven of these patients presented with mild symptoms, five remained asymptomatic, and none required hospitalization. Spencer et al. [19] enrolled 340 patients with PIBD and reported 51 (15%) infections with SARS-CoV-2, but no data regarding the clinical course of those patients were presented.

In a retrospective cohort study in an IBD referral center in Portugal, Μagalhaes et al. [20] reported 11 pediatric patients who tested positive for COVID-19; a total of 7 of them had CD and 4 had UC. The clinical course was either mild or totally asymptomatic, with mean symptom duration not exceeding 4 days and without need for hospitalization.

Sansotta et al. [22] surveyed 290 patients with PIBD and reported 25 cases of mild upper respiratory tract illness, presenting mainly with cough and fever, during a 4-month period. These cases were classified as suspicious for SARS-CoV-2 infection but due to the lack of widespread availability of nasopharyngeal tests, only two were laboratory confirmed.

In a prospective study conducted by Bosa et al. [15], 84 pediatric patients with IBD were followed from February 2020 to February 2021. During this period, 12 patients were diagnosed with COVID-19 (9 suffering from CD, 1 from UC, and 2 from IBD-U); a total of 4 were asymptomatic, while the remaining presented mild symptoms and none needed hospitalization.

Dolinger et al. [23] reported a case of a 14-year-old boy recently diagnosed with CD admitted to the hospital for COVID-19. His clinical course was severe with fever, gastrointestinal and respiratory symptoms, and rash. Laboratory tests revealed high inflammatory markers. He was treated with infliximab in an effort to co-manage PIBD exacerbation and suspected MISC-C, leading to resolution of symptoms.

### 3.3. IBD Disease Activity and COVID-19

Brenner et al. [13] reported that of 209 patients with PIBD enrolled in their study, 5% (*n* = 10) had severe IBD disease activity and 59% (*n* = 123) were in remission. Out of the 14 children who needed hospitalization, 14% (*n* = 2) had severe IBD activity and 50% (*n* = 7) had moderate IBD activity, while only 1 patient was in remission (7%). On the other hand, 63% of the 123 outpatients were in remission, while 4% (*n* = 8) had severe IBD activity.

In a cohort study from Italy [16] investigating the course of 185 patients with PIBD, 4 cases of SARS-CoV-2 infection were reported. Half of the patients exhibited mild symptoms, while the remaining were asymptomatic. Regarding IBD activity, two were in clinical remission and two had mild disease.

Ruan et al. [8] included 14 PIBD patients diagnosed with COVID-19. Out of them, 57% were in remission, while the rest had active IBD disease. Half of the patients exhibited symptoms such as fever, sore throat, headache, and fatigue, while the rest remained asymptomatic. No hospitalization attributed to COVID-19 was reported.

Bosa et al. [15] reported that of the 84 patients with PIBD enrolled in their study, 12 tested positive for SARS-CoV-2 (2 with active mild CD and the rest in remission).

### 3.4. IBD Medication and COVID-19

The data on the impact of medications used to treat PIBD on COVID-19 prognosis were often conflicting, indicating large heterogeneity in findings. Arrigo et al. [12] reported only 6 out of 2291 patients with PIBD tested positive for COVID-19, and only 1 of them experienced COVID-19-related pneumonia requiring hospitalization. This patient was being treated with azathioprine. No conclusions could be drawn about the putative causal relationship between the treatment and the course of infection, as three out of the remaining five patients (who exhibited mild symptoms) were also treated with azathioprine.

Ιn the aforementioned study from Portugal [20], aminosalicylates were the most commonly used treatment agents (*n* = 5, 45%). Other treatment regimens included anti-TNF (*n* = 2) and thiopurines (*n* = 2), either as monotherapy or combined (*n* = 2). Only one patient was treated with steroids. No association between treatment regimen and outcomes was noted, as all patients had mild symptoms not requiring hospitalization. Similarly, in two additional observational studies, enrolling 10 patients with PIBD and COVID-19, no differences in the clinical outcome were noted between children on different categories of immunomodulatory medication, as all patients presented with mild symptoms [22,24].

Brenner et al. [13] in their study (*n* = 209) reported that the most commonly prescribed IBD therapy was anti-TNF (*n* = 100, 48%), followed by sulfasalazine/mesalamine (*n* = 49, 23%). They noted that anti-TNF led to a decreased chance of hospitalization (51% of outpatients vs. 1% of hospitalized patients that received anti-TNF), while children who required hospitalization were mostly under treatment with sulfasalazine/mesalamine (57%) and steroids (29%). Accordingly, in a series of 291 pediatric patients with IBD reported by D’Arcangelo et al. [16], 81% (*n* = 149) were treated with infliximab; a total of 4 patients contracted SARS-CoV-2 (all treated with infliximab) with favorable outcome and no need for hospitalization.

In a single center cohort study conducted at Texas Children Hospital [8], 14 patients with PIBD under various treatment regimens who tested positive for SARS-CoV-2 were enrolled; a total of 50% developed mild symptoms and none required hospital admission. A total of 43% (*n* = 6) received monotherapy with biologic agents. No conclusions could be drawn regarding an association between patients’ therapy and clinical course of COVID-19.

Similarly, a retrospective study conducted in New York City [19] including 51 pediatric patients with IBD and COVID-19 with favorable outcome reported that 76% of patients received biologic agents.

Bosa et al. [15] reported that out of 12 patients with PIBD and COVID-19 included in their study, 2 received anti-TNF agents, 6 were on azathioprine, and 4 were on steroids. None of these patients required hospitalization. Two thirds had a mild course of COVID-19, while the other third remained asymptomatic.

Dolinger et al. [23] reported an intriguing case of a patient recently diagnosed with CD who was hospitalized due to severe COVID-19 infection and exacerbation of CD. He developed fever, rash, abdominal pain, tachycardia, and hypotension. He was treated for COVID-19 with azithromycin and hydroxychloroquine without improvement. Upon initiation of anti-TNF (infliximab), the patient’s symptoms resolved within hours.

## 4. Discussion

This systematic review aimed at investigating the existing literature regarding patients with PIBD and COVID-19. The prevalence of COVID-19 was 9.7%; a total of 5,9% of patients required hospitalization with a large variability across the studies (0–16.7%), and only 0.6% (0–1%) were admitted to the pediatric intensive care unit (PICU). Interestingly, no death was reported. The most common symptoms observed were cough, rhinitis, and fatigue, with 21% (0–25%) presenting gastrointestinal symptoms such as nausea, vomiting, and diarrhea. These findings are consistent with Mantovani et al. [3], who reported that in general, children and adolescents experience a mild course of COVID-19 and have good prognosis compared to adults. Tripothy et al. [25] in a meta-analysis on the outcomes of adult patients with IBD and COVID-19 reported that 33% required hospitalization, 4% admission to the ICU, and an overall mortality rate of 2.5%. The original concern that patients with PIBD are prone to more severe clinical course and progression of the disease does not seem to be supported by our review, in line with the currently available data on adults and children unaffected by IBD.

Our knowledge on the pathophysiology of COVID-19 continues to evolve and several mechanisms governing host–virus interaction have been proposed. The angiotensin converting enzyme 2 (ACE2) and the receptor of advanced glycation end-products (RAGE) pathways have been extensively studied and seem to play a pivotal role in the regulation of host inflammatory response and greatly impact disease severity. The ACE2 receptors at the epithelial cells of the terminal ileum and colon, lungs, kidneys, and blood vessels have been now established as the functional host receptors through which SARS-CoV-2 enters the cells and causes inflammation and multi-organ failure [26]. ACE2 receptor is highly expressed in the gastrointestinal tract, explaining why this system is commonly affected [9]. Apart from a binding receptor of SARS-CoV-2, ACE2 is a key regulator of the renin-angiotensin system (RAS), which is responsible for blood pressure and inflammation control. COVID-19 results in RAS dysregulation and impacts host inflammatory response. Additionally, recent clinical and experimental data have highlighted the role of RAGE activation in chronic inflammatory disease. In patients with IBD, upregulation of RAGE in the inflamed intestine has been reported [27]. Current data suggest that the activation of RAGE axis and dysregulation of RAS may be attenuated by a concurrent increase in circulating soluble forms of RAGE and ACE2, thus dampening the overall inflammatory response [28]. The aforementioned mechanisms could account for the lack of increased disease severity in patients with IBD and COVID-19.

Due to the scarce descriptive data available and lack of studies designed to infer causality from observational evidence, no reliable conclusion could be drawn regarding the possible impact of different treatments for IBD on the clinical course of COVID-19. Studies conducted in adult patients with IBD and COVID-19 demonstrate an association between IBD treatment and severity of COVID-19. There was an increased risk of severe infection in those under treatment with 5-aminosalicylic acid (5-ASA) or corticosteroid. Based on the SECURE-IBD database, systemic corticosteroid treatment has been linked to severe COVID-19 and death [13]. On the contrary, patients receiving anti-TNF were reported to exhibit mild symptoms [29]. There was no apparent harmful association between anti-TNF treatment and severe COVID-19, ICU admission, or death [13,17]. All the above conclusions are drawn from the meta-analysis of Tripathi et al. [25] in adult patients with IBD and COVID-19, suggesting that the use of biologics leads to a favorable outcome due to reduction of cytokine storm. In line with this finding, a very recent protocol for a phase-2 randomized controlled trial for testing the efficacy and safety of infliximab in treating COVID-19 has been published [30].

The presence of other comorbidities increases the overall risk of a severe clinical course of COVID-19 in PIBD patients. Brenner et al. [14] presented detailed characteristics of 14 COVID-19 patients, 7 out of whom had comorbidities, and 2 required PICU admission. Moderate or severe IBD activity and the presence of gastrointestinal symptoms were identified as additional risk factors [8,14].

MIS-C was initially described by The Royal College of Pediatrics and Child Health as a pediatric multisystem inflammatory syndrome temporarily associated with COVID-19 (PIMS-TS) [4]. Presenting symptoms include persistent fever, rash, abdominal pain, renal and cardiac dysfunction, conjunctivitis, coagulopathy, and, in some cases, shock requiring admission to the intensive care unit (ICU) [4,19,31]. The gastrointestinal tract is commonly affected, but the cause remains unknown, while a universal definition for MIS-C is still pending, and rapid evolution on the understanding of its pathophysiological mechanisms and treatment possibilities is gradually achieved [19,20,32]. Interestingly, there have been reported MIS-C cases where abdominal imaging reveals inflammation of the bowel wall in the distal ileum and mesenteric fat, a usual finding in IBD cases [33]. The overlapping symptoms of PIBD exacerbation and MIS-C pose a challenge for accurate diagnosis and decision on the appropriate treatment [23,33,34,35]. Physicians should be aware of the clinical presentations of both entities in order to provide the best available care to their patients.

The fear of COVID-19 and its association with other psychological and health outcomes in patients with IBD has been widely investigated. Emotional stress is a compounding factor for the symptoms of patients with IBD and could influence the severity of the disease [36]. Deterioration of health-related quality control in patients with PIBD was reported in the period during and after the quarantine for COVID-19, leading to an increased number of patients with active disease, as well as to reduced disease control [37]. However, the pandemic still presents a multifaceted challenge in the complex handling of a patient with PIBD. As presented by Martinelli et al. [21] in a cohort of 180 children with IBD observed during the lockdown, a significant reduction of IBD flare-related hospital admissions was reported, compared to the 2-month period prior to the pandemic, possibly indicating prioritization of reducing social contact over seeking timely medical treatment. In addition, a large percentage of patients’ parents expressed significant concern over continuing immunomodulatory maintenance treatment, with 18% postponing the infusion by their own volition and a further 6% opting for discontinuation, despite reassuring medical consultation. Bosa et al. [15], as well as Arrigo et al. [12], mentioned delays in planned infusions and in initiation of biologic therapy, which lead to modifications and delays in treatment course. Dorfman et al. [38] reported fear of severe infection in 81% of the enrolled PIBD patients, while 34% missed their regular appointments and 3% discontinued or changed their course of treatment, due to COVID-19. This reflects both the parental fear and the misinformation regarding the perceived increased risk of infection in PIBD patients. The data derived from adult populations also suggest that COVID-19 increased anxiety in patients with IBD and imposed fear that their medication will worsen a possible infection [39]. ESPAGHAN [24] recommends continuing the indicated treatment, in order to achieve remission and avoid delays in the initiation of therapy. It is important to highlight that suspending therapy may lead to an IBD exacerbation or the formation of antibodies, consequences far more worrying than a possible SARS-CoV-2 infection. The currently available data does not suggest an increased risk of severe COVID-19 in patients with PIBD. Conversely, adherence to the medication regimen is pivotal for IBD control [40]. This was also noted in adult patients, as achieving IBD remission remains a priority [25].

The expedited development and authorization of vaccines aiming at the SARS-COV2 virus was a step of paramount importance towards reducing disease-associated morbidity and mortality worldwide. Even though initial trials excluded IBD patients due to their immunosuppressed status, satisfactory efficacy and tolerance of vaccination in this population has since been described in various studies [41,42,43]. According to small sample observational studies enrolling PIBD patients, no serious adverse effects were reported [44,45]. The majority exhibited local symptoms, with systemic adverse events being transient and mild and no reports of serious complications such as myocarditis. Seroconversion rates after vaccination were comparable to the control group. Conversely, neutralizing antibody responses in children receiving combination therapy (anti-TNFa and immunomodulators) were reduced [45], while in other studies, monotherapy with anti-TNFa was associated with decreased levels of anti-receptor binding domain IgG antibodies compared to immunomodulators alone [46]. Hence, expert guidelines released by various gastroenterological societies have advised in favor of early administration of mRNA vaccines [47,48]. However, the literature data regarding the effect of vaccination on patients with PIBD remain scarce and future large-scale studies are warranted in order to draw valid and safe conclusions on the overall protective and immunogenic role of SARS-CoV2 vaccines on this particular patient population, as well as any potential interactions with specific therapeutic regimens.

Despite ongoing pro-vaccination campaigns aiming particularly at high-risk groups, skepticism of a considerable portion of the adult IBD population towards vaccines has been well documented in various studies. This hesitancy is a complex phenomenon affected by multiple variables, ranging widely among different subpopulations and countries, while standing as a possible obstacle on the road to achieving herd immunity. Sociodemographic factors reportedly associated with increased reluctance towards vaccination include lower income, younger age, geographic location (e.g., certain Middle Eastern regions) and even the presence of specific chronic conditions (e.g., autoimmune diseases) [49,50]. Hesitancy or complete rejection of COVID-19 vaccination varied widely across studies conducted around the world, ranging from 17.7 % to 50.7%. Most individuals cited fear of adverse effects and concerns about compromised vaccine safety due to the unprecedented speed of vaccine development as inhibiting factors, expressing their desire for more extensive evidence on the efficacy of all available vaccines and their respective safety profiles [51,52,53]. Although, there are no data regarding the attitudes of parents of children with IBD towards vaccination for SARS-CoV-2, studies have reported that parents of children with other chronic diseases, such as asthma, demonstrated higher vigilance to vaccinate their children against COVID-19 [54].Considering that vaccination of the pediatric population is dependent on the parents’ judgment, scientists have attempted to determine possible factors affecting their attitude and skepticism towards the available vaccines. The modified Vaccine Hesitancy Scale (VHS), a verified tool developed by WHO Strategic Advisory Group of Experts on Immunization (SAGE), has been used to quantify the levels of parental uncertainty [55,56]. Interestingly, increased reluctance has been observed concerning COVID-19 vaccines, in comparison to routine childhood vaccinations [55,56]. This may be attributed to apprehension due to rapid vaccine development and deployment, as well as the perceived low risk since infection usually runs a milder course in younger patients. The role of social media in shaping public opinions is being increasingly recognized; even though recent experience during the pandemic has been unfavorable with various platforms being used as a breeding ground for spreading potentially harmful and malignant misinformation [57,58,59], if used currently, they could prove very valuable as a means of raising awareness and providing access to valid medical knowledge concerning the importance of vaccination. In these extraordinary circumstances, organized educational campaigns and the active involvement of healthcare professionals seem crucial in order to optimize vaccine acceptance and increase vaccination rates across all ages.

## 5. Conclusions

Our systematic review of the literature revealed that the prevalence of COVID-19 in patients with PIBD is low and that, despite initial concerns, no convincing evidence exist regarding an increased disease severity in these vulnerable patients. However, the pandemic still presents a multifaceted challenge in the complex handling of patients with PIBD. Long-term studies are needed to elucidate the pathophysiologic mechanisms governing host response to infection in populations with specific co-morbidities under various treatment regimens.

## Figures and Tables

**Table 1:** Characteristics of studies included in the systematic review reporting data on the occurrence of COVID-19 in pediatric patients with IBD.

Author	Country	Total IBD Patient (No)	Total IBD Patient with COVID-19 (No)	No Need for Hospitalization (No)	Hospitalization (No)	ICU Admission (No)	Deaths (No)	CD (No)	UC (No)	Mean/ Median, Age (yrs)	Mean Outcomes
Arrigo et al., 2020, [12]	Italy	2291	6	5	1	0	0	2	4	13.5	Mild symptoms—1 severe infection
Brenner et al., 2021 [13]	23 countries—USA, France, Italy, Argentina, UK,	209	209	195	14	2	0	138	61	15/16	Mild symptoms—14 hospitalized—2 PICU
Brenner et al., 2020 [14]	33 countries	29	29	26	3	0	0	N/A	N/A	0–19	Mild symptoms—3 severe infection
D’Arcangelo et al., 2021 [16]	Italy	185	4	4	0	0	0	2	2	N/A	2 asymptomatic—2 mild symptoms
Bosa et al., 2022 [15]	Italy	84	12	12	0	0	0	9	1	14/1–18	4 asymptomatic—8 mild symptoms
Koletzko et al., 2021 [17]	Germany	89	25	25	0	0	0	44	34	6–20	Mild symptoms
Queiroz et al., 2021 [18]	Latin America (13 countries)	18	18	17	1	0	0	N/A	N/A	0–18	Mild symptoms
Dolinger et al., 2020 [23]	USA	1	1	0	1	0	0	1	0	14	Severe symptoms
Spencer et al., 2021 [19]	USA	340	51	N/A	N/A	0	0	39	9	0–21	N/A
Ruan et al., 2021 [8]	USA	14	14	14	0	0	0	10	4	N/A	7 transient symptoms—5 asymptomatic
Μagalhaes et al., 2021 [20]	Portugal	268	11	11	0	0	0	7	4	15 (7–18)	100% mild disease—no complication
Turner et al., 2020 [24]	Portugal-Global study	8	8	8	0	0		5	2	N/A	100% mild disease—no complication
Martinelli et al., 2020 [21]	Italy	180	0	0	0	0	0	0		0	No COVID-19 cases
Sansotta et al., 2021 [22]	Italy	290	2	2	0	0	0	0	2	15.2 (2–18)	100% mild disease—no complication

Abbreviations: N/A, not available; IBD, inflammatory bowel disease; ICU, intensive care unit; CD, Crohn’s disease; UC, ulcerative colitis.

**Table 2:** Studies that provide data regarding patients with PIBD hospitalized due to COVID-19.

	Arrigo et al., 2020, [12]		Brenner et al., 2020 [14]		Brenner et al., 2021 [13]				Dolinger et al., 2020 [23]		Queiroz et al., 2021 [18]		
Total IBD Patient (No)	2291		29		209				1		18		
Total IBD patient with COVID-19 (No)	6		29		209				1		18		
No need for hospitalization (No)	5		26		195				0		17		
Hospitalization (No)	1		3		14				1		1		
	IBD activity	Treatment	IBD activity	Treatment	IBD activity		Treatment		IBD activity	Treatment	IBD activity	Treatment	
	remission	azathioprine	N/A	N/A	mild	3	Sulfasalazine/mesalamine	8	severe	infliximab	N/A	N.A	
					moderate	8	Steroids	4					
					severe	1	6MP/azathioprine monotherapyc	2					
					remission	1	Methotrexate monotherapyc	0					
					unknown	1	TNF antagonist	1					
							TNF antagonist/ 6MP/AZA/MTX	2					
							Anti-integrin (vedolizumab)	2					
							IL12/23 inhibitor	2					
							Janus kinase inhibitor	0					
							Other IBD medication(s)	1					
							No IBD medication	1					
ICU admission (No)	0		0		2				0		0		
	IBD activity	Treatment	IBD activity	Treatment	IBD activity		Treatment		IBD activity	Treatment	IBD activity		Treatment
	-	-	-	-	unknown	1	Sulfasalazine/Mesalamine	2	-	-	-		-
					moderate	1							

Abbreviations: N/A, not available; IBD, inflammatory bowel disease; ICU, intensive care unit.

## Data Availability

Data is contained within the article.
